# Supplementary figures and images for: Characterizing eye-gaze positions of people with severe motor dysfunction: Novel scoring metrics using eye-tracking and video analysis
Source: PLoS One. 2022 Aug 31;17(8):e0265623. doi: 10.1371/journal.pone.0265623 (PMC9432701; doi:10.1371/journal.pone.0265623)

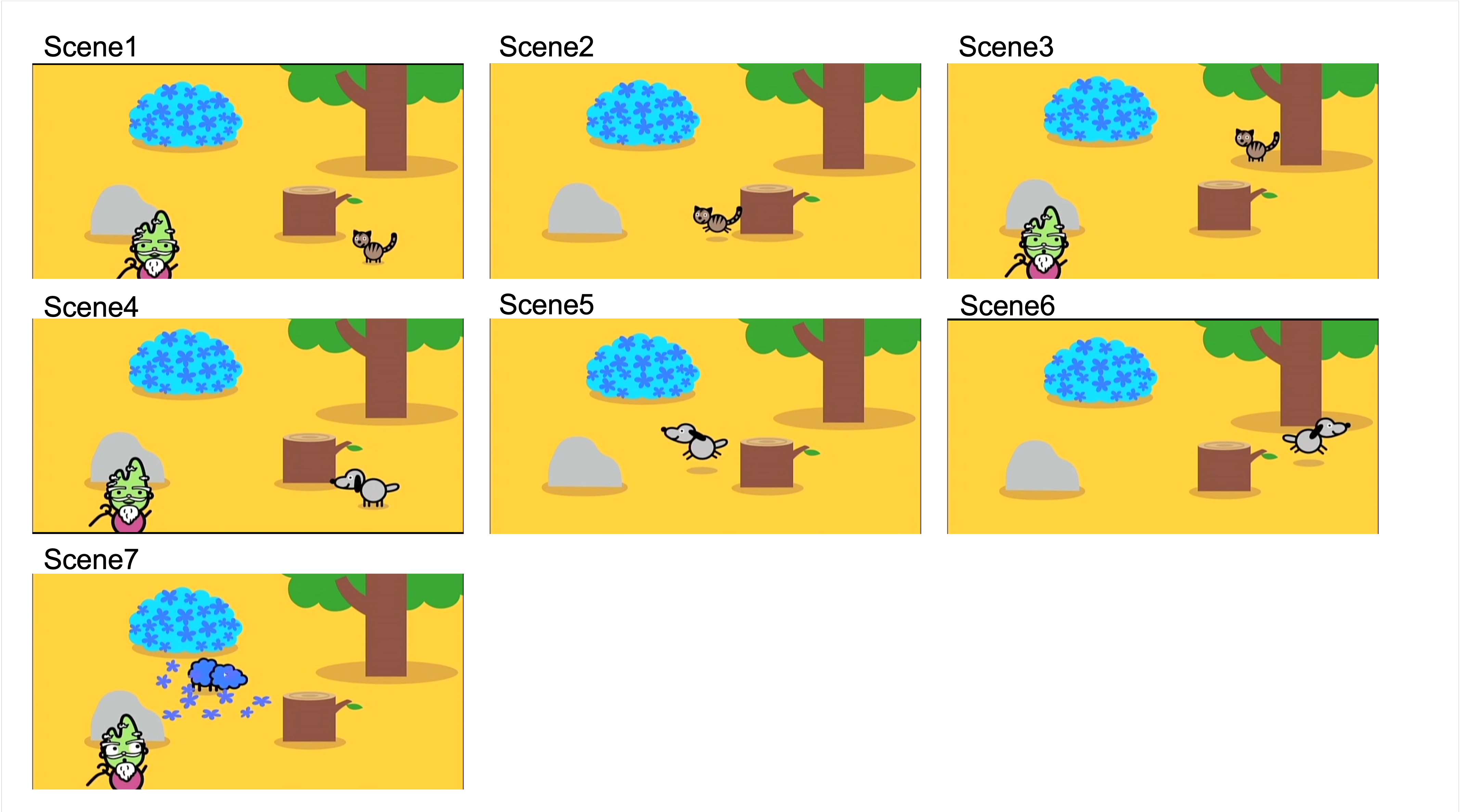

Supplement: S1 Video — (TIFF) [file pone.0265623.s001.tiff]

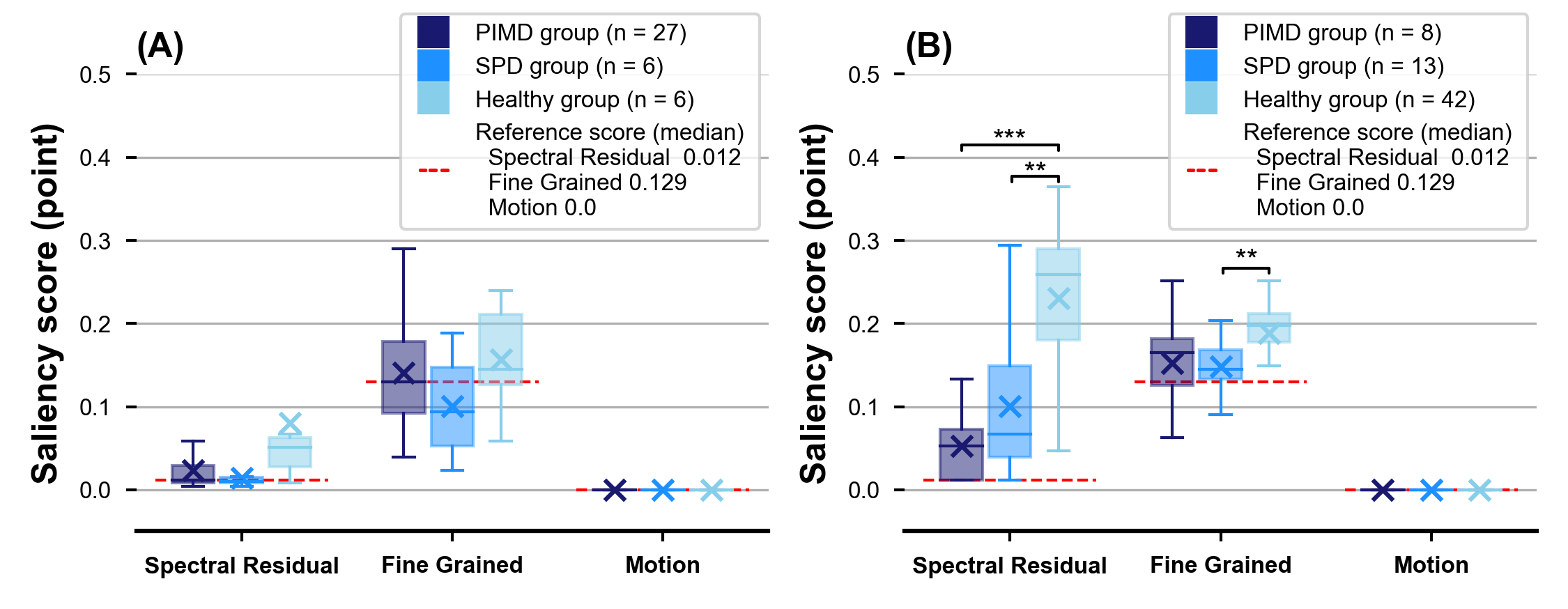

Supplement: S1 Fig — Distribution of saliency scores for the entire video for the PIMD, SPD, and healthy groups are presented as box-and-whisker plots, under the conditions of eye-gaze acquisition time of (A) < 26.1 s and (B) ≥ 26.1 s. Box-and-whisker plots show median values (−), average (×), interquartile ranges, minimum and maximum values, and outliers. *p < .05, **p < .01, ***p < .001. PIMD, profound intellectual and multiple disabilities; SPD, severe physical disabilities. (TIF) [file pone.0265623.s002.tif]

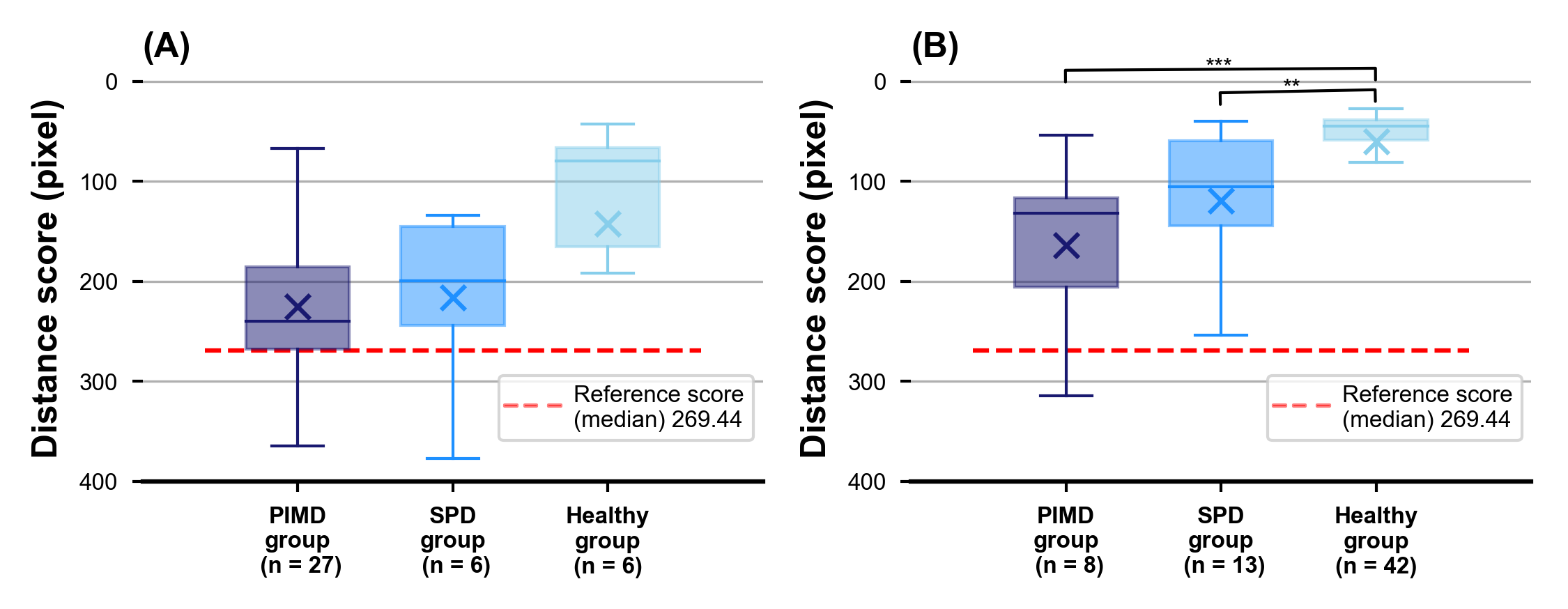

Supplement: S2 Fig — Distribution of distance scores for the entire video for the PIMD, SPD, and healthy groups are presented as box-and-whisker plots, under the conditions of eye-gaze acquisition time of (A) < 26.1 s and (B) ≥ 26.1 s. Box-and-whisker plots show median values (−), average (×), interquartile ranges, minimum and maximum values, and outliers. *p < .05, ** p < .01, ***p < .001. PIMD, profound intellectual and multiple disabilities; SPD, severe physical disabilities. (TIF) [file pone.0265623.s003.tif]

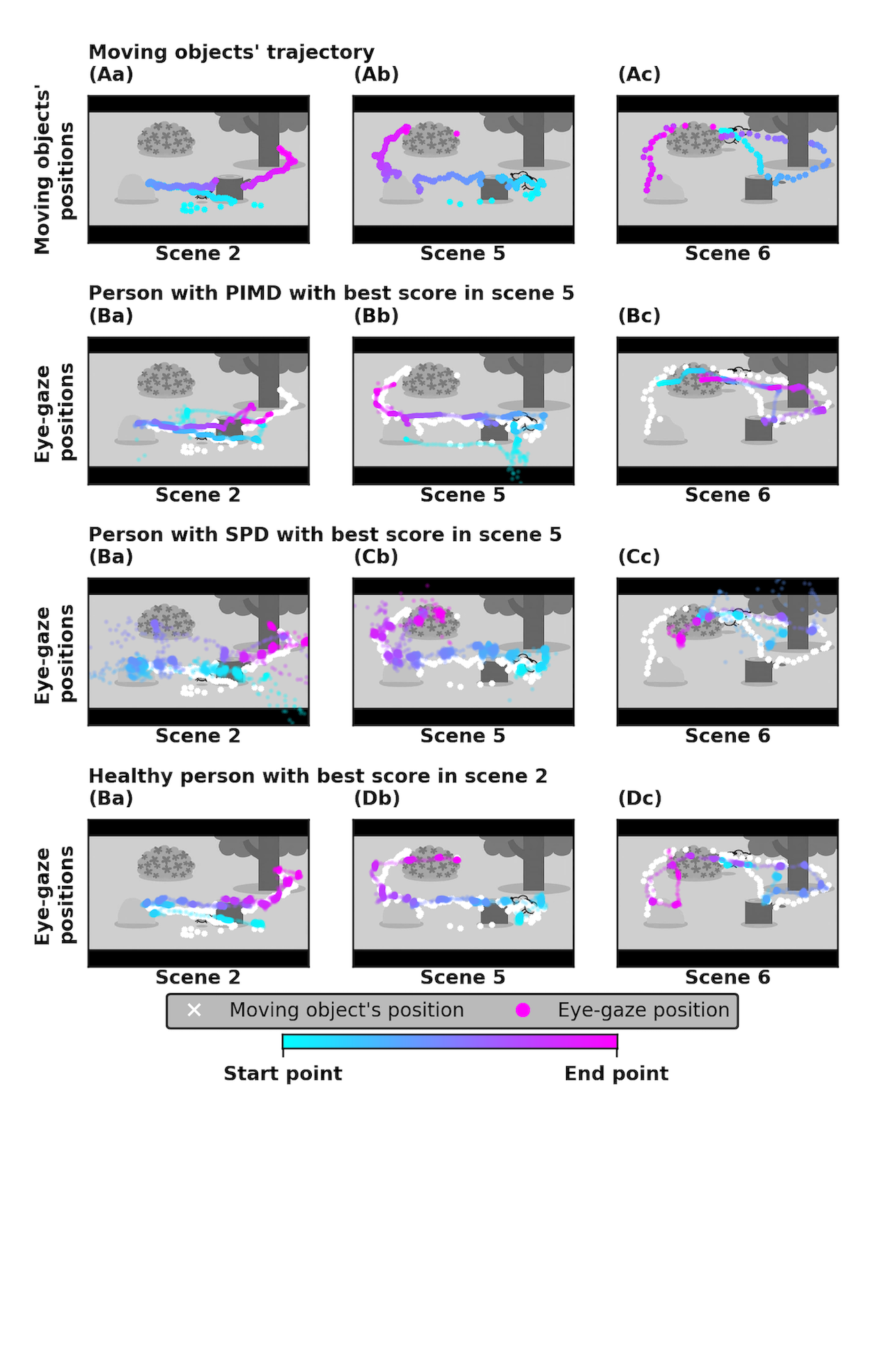

Supplement: S3 Fig — Moving objects’ position (x) and eye-gaze position (o) were plotted in (a) scene 2, (b) scene 5, and (c) scene 6 for representative subjects. A color gradient represents the start (blue) and the end (red) points of the scene. (A) A moving objects’ trajectory. (B) Eye-gaze positions of a person with PIMD who had the best distance score for scene 5. (C) Eye-gaze positions of a person with SPD who had the best distance score for scene 5. (D) Eye-gaze positions of a healthy subject who had the best score for scene 2. (TIF) [file pone.0265623.s004.tif]
