# Supplementary material for: Characterizing eye-gaze positions of people with severe motor dysfunction: Novel scoring metrics using eye-tracking and video analysis
Source: PLoS One. 2022 Aug 31;17(8):e0265623. doi: 10.1371/journal.pone.0265623 (PMC9432701; doi:10.1371/journal.pone.0265623)
Supplement: S1 Table — (DOCX) [file pone.0265623.s005.docx]

**S1 Table. Specifications of Tobii Pro^®^ Spectrum eye-tracking device**

| **Eye tracking specifications** | **Description** |
| --- | --- |
| **Accuracy** | 0.3° at optimal conditions |
| **Total system latency** | Less than 3 frames |
| **Blink recovery time** | 1 frame |
| **Gaze recovery time** | Less than 150ms |

Tobii Pro Spectrum. Available from https://www.tobiipro.com/product-listing/tobii-pro-spectrum/.
