# Supplementary material for: Characterizing eye-gaze positions of people with severe motor dysfunction: Novel scoring metrics using eye-tracking and video analysis
Source: PLoS One. 2022 Aug 31;17(8):e0265623. doi: 10.1371/journal.pone.0265623 (PMC9432701; doi:10.1371/journal.pone.0265623)
Supplement: Attachment — Submitted filename: Response_to_reviewers.docx [file pone.0265623.s006.docx]

**Reviewer #1:**

***Response:***

*We appreciate the time and effort the reviewer has dedicated to providing insightful feedback on ways to strengthen our paper. We have incorporated changes that reflect the detailed suggestions you have graciously provided. We hope that our edits and the responses we provide below satisfactorily address all the concerns the reviewer has noted.*

Dear Authors, the article in general sounds correct, but I have found a few unaddressed issues:

*We deeply appreciate for your positive feedback.*

1. Did you consider the system’s accuracy? According to the producer’s website, the highest accuracy at optimal conditions is 0.3 degrees. That is almost 4 mm at a 65 cm distance, it might be possible that in your case, the error is much higher (were “optimal conditions” assured?). How does it affect the final results?

*Thank you for pointing out a crucial issue. As pointed out by the reviewer, the accuracy and measurement conditions of eye tracking are essential issues when using eye-tracker devices. In this study, a single observer performed measurements using the same device and video to minimize the measurement error on the observer side. On the other hand, the measurement conditions of the subject differed from person to person because each subject had a different clinical background and spent leisure time differently. Therefore, there may be measurement errors for each subject as well as the device accuracies.*

*So far, no studies have addressed the objective evaluation of the gaze characteristics of people with SPD and PIMD. Although the limitation of measurement errors, the present study has, for the first time, succeeded in obtaining and evaluating the eye-gaze positions and patterns of people with SPD and PIMD, which showed the significance of our novel scoring metrics using eye-tracking and video analysis. We added following sentences on measurement errors to paragraph of the Strengths and limitations.*

Line 456–459:

First, there were variations in the clinical backgrounds, comorbidities, visual acuity, and experience with viewing videos. Second, measurement errors for each subject and device accuracy might occur because of the difference in individual measurement conditions.

2. There is no information about the tracking system latency.

*We apologize for the insufficient description. The tracking system latency of the device is 50 µs which is less than timestamps acquisition duration (1670 µs). We have added the manuscripts in the paragraph of Method section as follows, in accordance with the reviewer’s suggestion.*

Line 108–109:

The tracking system latency of the device was 50 µs which is less that sampling frequency.

Did you perform a temporal calibration? Is the offset constant?

*Thank you for pointing out an essential issue. It is not easy to perform accurate calibration of eye-gaze positions in people with SPD and PIMD as they are usually hard to follow the instructions. Moreover, it would be a burden on patients to continue the experiment until accurate calibration becomes possible. Therefore, in people with SPD and PIMD, calibration was performed by a healthy person instead of each subject. Although calibration can affect the accuracy and offset, as the reviewer is concerned, we believe that this does not affect the importance of the main findings of this study.*

3. Could you elaborate on whether the moving object’s position should be defined as the object’s center of gravity? I can imagine the situation that the watcher is staring at an eccentric part of an object all the time, e.g., the dog’s eye or nose.

*Thank you for pointing out fundamental issues related to the aim of our study. We concluded that the object's center of gravity was appropriate for the following three reasons:*

1. *The aim of the study was to establish novel metrics to analyze the eye-gaze pattern between the objects and the eye positions.*
2. *The distance between the eyes and the mouth of the character is so close (the distance was around 15 pixels on 480 x 720 pixels screen) that exact distance between the eccentric part in the characters and eye gaze position was not accurately acquired.*
3. *Observation of people with SPD and PIMD revealed that the places where they focus their attention were so diverse that it is difficult to unify them to a specific object.*

*The present study used the video, in which the characters move well in the field of view, to analyze the eye-gaze position and patterns for the moving objects. To clarify the cognitive functions such as staring at distinct parts of the face, as the reviewer suggested, a future investigation may be necessary to conduct using video images focused on the specific area of the objects (e.g., a face with a close-up of the eyes and mouth) that are large enough to evaluate which feature areas are being viewed by the subjects.*

**Reviewer #2:**

***Response:***

*We appreciate the time and effort the reviewer has dedicated to providing insightful feedback on ways to strengthen our paper. We have incorporated changes that reflect the detailed suggestions you have graciously provided. We hope that our edits and the responses we provide below satisfactorily address all the concerns the reviewer has noted.*

The research presents a quantified approach for assessment of gaze for people with motor disabilities. The research can be extended to analyze gaze of any individual based on saliency score.

The language of presentation is clear.

The data analysis in the present work show difference of saliency score between healthy individuals and people with MD.

However the technique of video analysis (technique of calculating scoring metrics between video frames) seems cumbersome.

*We sincerely appreciate your positive feedback. The saliency map for calculating the saliency score used in this study is based on a standard tool (OpenCV) and can be calculated with simple process. The distance score can also be easily measured from the*

*center of gravity of the moving object and the eye-gaze position.*

*We have disclosed all codes for scoring metrics which are available from https://github.com/clinfo/TobiiEyeTrackerVideoAnalysis.git.*
