# Supplementary material for: Characterizing eye-gaze positions of people with severe motor dysfunction: Novel scoring metrics using eye-tracking and video analysis
Source: PLoS One. 2022 Aug 31;17(8):e0265623. doi: 10.1371/journal.pone.0265623 (PMC9432701; doi:10.1371/journal.pone.0265623)
Supplement: Attachment — Submitted filename: Response_to_reviewers.docx [file pone.0265623.s007.docx]

**Reviewer #1:**

***Response:***

*We appreciate the time and effort the reviewer has dedicated to providing insightful feedback on ways to strengthen our paper. We have incorporated changes that reflect the detailed suggestions you have graciously provided. We hope that our edits and the responses we provide below satisfactorily address all the concerns the reviewer has noted.*

Dear Authors, I appreciate your answers. Unfortunately, my concerns were not addressed in the paper, and you took just a minimal effort to improve its quality. I cannot agree that adding three simple sentences is a sufficient improvement after a major review decision. Therefore I assume the paper still needs to be significantly revised.

*We apologize for the insufficient description to reflect the insightful feedback of the reviewer. In the previous revision, the reviewer pointed out the following concerns:*

*1) Details about the system’s accuracy and its impact on the results*

*2) Information on the tracking system latency, temporal calibration, and offsets.*

*3) Whether the moving object’s position should be defined as the object’s center of gravity?*

*We have newly addressed the concerns that the reviewer graciously provided by adding the following paragraph and the table to the Discussion section.*

**Advantages and limitations of the eye-tracker device in evaluating the gaze responses of people with SPD and PIMD**

It is challenging to evaluate eye-gaze responses in people with SPD and PIMD with the same accuracy as healthy subjects. The most confounding factors affecting accuracy are as follows: 1) erratic eye movements are often observed in people with PIMD so that the gaze position shifts with instantaneous amplitude, 2) people with SPD and PIMD are usually hard to follow instructions, 3) people with SPD and PIMD have difficulty maintaining interest in particular objects and need to complete the measurements in a short time. Therefore, to analyze gaze positions in SPD and PIMD patients, eye-tracking devices with high sampling frequency and short, objective observations in an environment suitable for measurement are essential to ensure accuracy. Although eye-tracking could be difficult for conventional devices with low sampling frequency, we used Tobii Pro® Spectrum eye-tracking device to analyze eye-gaze positions in the present study. The tracking system latency of the device was 50 µs that enabled high sampling frequency faster than erratic eye movements (S1 Table).

S1 Table. Specifications of Tobii Pro® Spectrum eye-tracking device

| **Eye tracking specifications** | **Description** |
| --- | --- |
| **Accuracy** | 0.3° at optimal conditions |
| **Total system latency** | Less than 3 frames |
| **Blink recovery time** | 1 frame |
| **Gaze recovery time** | Less than 150ms |

Tobii Pro Spectrum. Available from https://www.tobiipro.com/product-listing/tobii-pro-spectrum/.

Temporal calibration and offsets of eye-gaze positions in people with SPD and PIMD are matters, as people with SPD and PIMD have difficulty looking at the instructed points. Therefore, calibration and offset settings were performed by a healthy person instead of each subject. Intergroup comparisons were carried out to reduce measurement error due to the device. In addition, a single observer performed measurements using the same device and video to minimize the measurement time and errors on the observer side. On the other hand, the measurement conditions of the subject differed from person to person because each subject had a different clinical background and spent leisure time differently. So far, no studies have addressed the objective evaluation of the gaze characteristics of people with SPD and PIMD. Although the limitation of measurement errors, the present study has, for the first time, succeeded in obtaining and evaluating the eye-gaze positions and patterns of people with SPD and PIMD, which showed the significance of our novel scoring metrics using eye-tracking and video analysis. The present study was designed to analyze the eye-gaze position and patterns of moving objects that attracted the subject’s interests. The cognitive functions were not addressed, as a distinct position in the face was so close (e.g., the distance between the eye and mouth was about 15 pixels on a 480 x 720-pixel screen) that precise evaluation of what were stared had not been performed precisely. A future investigation may be necessary to conduct using video images focused on the specific area of interest in the objects.

**Reviewer #2:**

The metrics explained in the manuscript might be helpful for models that are being developed for people with disabilities. The methodology specified might be helpful in gaze analysis. I find the manuscript ready for publication..

***Response:***

*We sincerely appreciate the time and effort the reviewer has dedicated and positive assessment of our research findings.*
